# Prevalence and Antimicrobial Susceptibility Pattern of Pseudomonas aeruginosa Isolates From Various Clinical Specimens in a Tertiary Care Hospital: An Analysis of Resistance Trends and Implications for Treatment Strategies

**DOI:** 10.7759/cureus.72556

**Published:** 2024-10-28

**Authors:** Tulika Harsh, Harsha V Patil, Satish R Patil

**Affiliations:** 1 Department of Microbiology, Krishna Institute of Medical Sciences, Krishna Vishwa Vidyapeeth (Deemed to be University), Karad, IND

**Keywords:** cefepime, hospital acquired infection, imipenem, multidrug resistant, pseudomonas aeruginosa

## Abstract

Background: *Pseudomonas aeruginosa*, an aerobic Gram-negative bacillus, is one of the primary causes of severe healthcare-associated infections, especially in people with compromised immune systems or those who are critically ill. It is common in patients with burn injuries, cystic fibrosis, and organ transplants, causing serious infections like pneumonia and septicemia. Carbapenem resistance, driven by mechanisms such as drug efflux and beta-lactamase production, including metallo-β-lactamases, poses a significant clinical threat. Multidrug-resistant (MDR) strains lead to nosocomial outbreaks with high morbidity and mortality. This study aims to assess the prevalence and resistance patterns of *Pseudomonas aeruginosa* to optimize treatments and inform antibiotic policies.

Material and methods: This cross-sectional study was conducted in the Department of Microbiology for a period of one year (November 2022 to November 2023). In total, 118 clinical specimens were processed for identification and antimicrobial sensitivity testing using the Kirby-Bauer disk diffusion technique, following the Clinical and Laboratory Standards Institute guidelines.

Result: Out of the 118 clinical samples, urine samples yielded the highest number of isolates (50%), followed by the pus samples (28.81%). Male patients accounted for 67.8% of the isolates, and female patients 32.2%. The highest prevalence was observed in the 41-60 age group, representing 33.90% of the cases. Eighty-seven percent of the isolates were from the inpatient department. In total, 28.86% of the isolates came from the surgery ward. *Pseudomonas aeruginosa* demonstrated the highest sensitivity to cefepime (31.3%), followed by amikacin (26.3%), and the slightest sensitivity to imipenem and piperacillin.

Conclusion: *Pseudomonas aeruginosa* is a frequently isolated organism and is becoming increasingly resistant to standard medications. Notably, aminoglycosides (such as amikacin) and cephalosporins (such as cefepime) demonstrated efficacy against *Pseudomonas*, though the frequency of MDR* *strains is rising. The current investigation provides valuable insights into the incidence of *Pseudomonas aeruginosa* and its susceptibility to various antibiotics, which will guide the adoption of improved patient care and infection control methods.

## Introduction

*Pseudomonas aeruginosa* is a non-fermentative, aerobic, Gram-negative bacillus and is one of the leading causes of severe healthcare-associated infections, especially in critically ill and immunocompromised patients [1-2]. As an opportunistic pathogen, it is capable of causing serious invasive diseases, and its infections are notably prevalent in individuals with burn injuries, individuals who have mucoviscidosis, and individuals who have had organ transplants [3]. The bacterium possesses an array of secretion systems that release proteins essential to the pathogenesis of clinical strains [4]. Severe infections caused by Pseudomonas include malignant external otitis, endophthalmitis, meningitis, pneumonia, and septicemia, often associated with resistance to carbapenem therapy, a major concern in treatment failure [3]. Carbapenem resistance observed in *Pseudomonas aeruginosa* is mediated by several mechanisms, which include drug efflux, modifications to or lack of proteins found on the outer membrane (such as OprD or Porin), alterations in membrane permeability, and the production of β-lactamases, particularly serine and metallo-β-lactamases (MBLs) [5]. The growing threat of multidrug-resistant (MDR) *Pseudomonas aeruginosa* is exacerbated by the limited development of new antimicrobial agents [6]. This study aims to assess the prevalence and resistance patterns of MBL-producing strains to improve therapies and antibiotic policies.

## Materials and methods

Study design, period, and sample size

The current cross-sectional study was carried out in the Department of Microbiology, Krishna Institute of Medical Sciences, Krishna Hospital and Medical Research Centre, Karad, from November 2022 to November 2023 on 118 *Pseudomonas aeruginosa* isolates. The Institutional Ethical Committee accepted the study, and ethical clearance (approval number KIMSDU/IEC/04/2022) was obtained from Krishna Institute of Medical Sciences (Deemed to be University), Karad, under protocol number 067/2021-2022.

Inclusion criteria

*Pseudomonas aeruginosa* isolates from various clinical samples of both sexes and all age groups received in the laboratory were included.

Exclusion criteria

The study excluded *Pseudomonas aeruginosa* isolates from the same patient’s specimen to prevent duplication.

Sample collection and processing

All the samples, including urine, sputum, pus, endotracheal tube, and body fluids, were subjected to Gram staining for microscopic examination and were cultured on suitable culture media, such as MacConkey Agar and Blood Agar, and incubated overnight at 37°C. *Pseudomonas aeruginosa* was identified based on its colony morphology, biochemical reactions, the oxidase test, and pigment production [7].

Antibiotic susceptibility testing

In compliance with the Clinical and Laboratory Standards Institute standards [8], antibiotic susceptibility testing was performed on Mueller-Hinton agar using the Kirby-Bauer disk diffusion method, utilizing commercially available antibiotic disks (HiMedia, Mumbai). *Pseudomonas aeruginosa* ATCC 27853 was used as the control strain. The antibiotics tested in our study were imipenem (10 μg), piperacillin-tazobactam (100/10 μg), levofloxacin (30 μg), gentamicin (10 μg), amikacin (30 μg), ciprofloxacin (30 μg), ceftazidime (30 μg), cefepime (30 μg), meropenem (10 μg), and piperacillin (100 μg).

Statistical analysis

The data were entered into the Microsoft Excel (Microsoft Corporation, Redmond, WA) spreadsheet. The results were presented as percentages, and tables were generated to serve different purposes.

## Results

A total of 118 samples were collected during the study period of one year. Out of the 118 isolates, male patients accounted for 67.8% and female patients 32.2%. The majority of the patients (33.90%) belong to the 41-60 age group, followed by the 21-40 and >60 age groups (32.2% each).

Among the male population, the majority of the isolates were from the 21-40 age group (23.72%), whereas the majority of the isolates among the female population were from the 41-60 age group (14.40%) (Table 1).

**Table 1:** Age- and gender-wise distribution of Pseudomonas aeruginosa n: number.

Age group	Male (n)%	Female (n)%	Total	Percentage
0-20	2 (1.69)	0	2	1.69%
21-40	28 (23.72)	10 (8.47)	38	32.20%
41-60	23 (19.49)	17 (14.40)	40	33.90%
>60	27 (22.88)	11 (9.32)	38	32.20%
Total (n)	80	38	118	100%

Out of the 118 isolates, 87% were from the inpatient department, and 13% were from the outpatient department.

**Figure 1:**
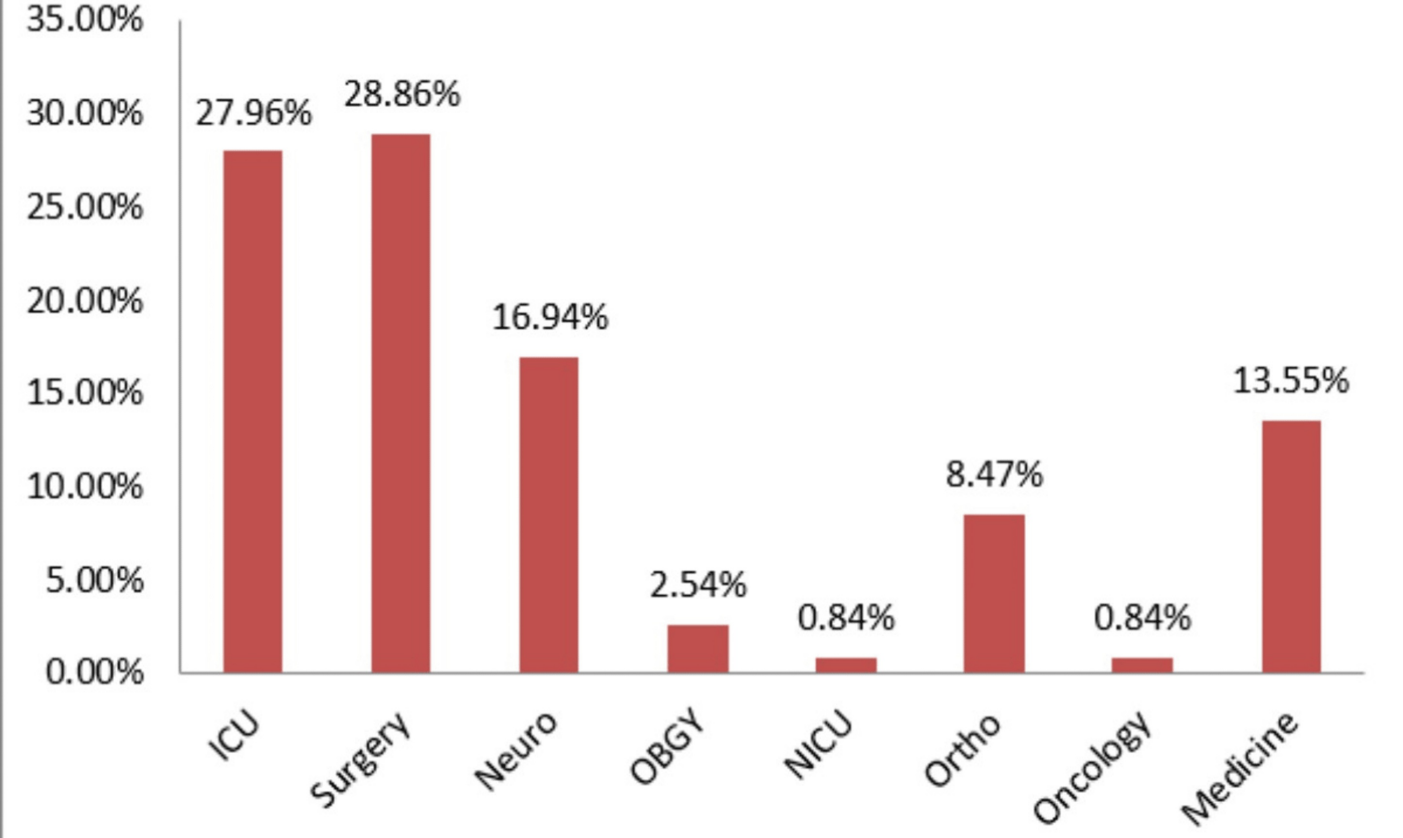
Ward-wise distribution of Pseudomonas aeruginosa ICU: intensive care unit, Neuro: neurology, OBGY, obstetrics and gynecology, NICU: neonatal intensive care unit, Ortho: orthopedic.

Ward-wise, the majority of the isolates were from the surgery ward (28.86%), followed by the intensive care unit (ICU; 27.96%), neurology (16.94%), medicine (13.55%), orthopedic (8.47%), obstetrics and gynecology (2.54%), and neonatal ICU and oncology (0.84% each) (Figure 1).

Out of the 118 samples, the majority of the isolates came from the urine samples (50%), followed by pus (28.81%), endotracheal tube (14.41%), body fluids (4.24%), and sputum (2.54%) (Table 2).

**Table 2:** Distribution of Pseudomonas aeruginosa among various clinical specimens

Specimens	Total	Percentage
Pus	34	28.81%
Urine	59	50%
Sputum	3	2.54%
Endotracheal tube	17	14.41%
Body fluids	5	4.24%
Total	118	100%

The isolates showed the highest sensitivity pattern to cefepime (31.3%), followed by amikacin (26.3%), gentamicin (19.50%), ceftazidime (16.1%), and piperacillin-tazobactam (8.5%).

**Figure 2:**
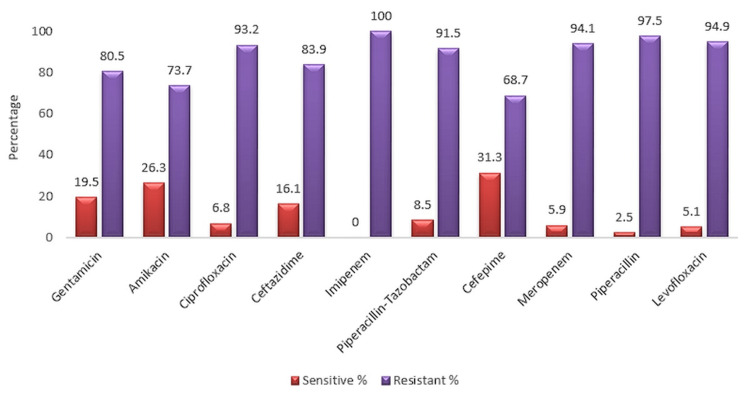
Antibiotic sensitivity profile of Pseudomonas aeruginosa

On the other hand, maximum resistance was noted with imipenem (100%), followed by piperacillin (97.5%), and levofloxacin (94.9%) (Figure 2).

## Discussion

Out of the 118 samples involved in the current study, the maximum number of isolates obtained was from the 41-60 age group (33.90%). The findings from numerous other studies align with our research. Sreeshma reported the highest isolation rate (34.70%) in the 41-60 age group [9]. Similarly, Shyamasree found 25.33% of isolates in the same age group [10]. Jayanthi also observed a peak in isolation (30.52%) in this group [11]. Additionally, Chauhan (35%), Chourasia (34.35%), and Andhale (46%) reported similar trends in the 41-60 age group [12-14].

The majority of the *Pseudomonas aeruginosa* isolates were obtained from male patients (67.80%), compared to female patients accounting for 32.20%. A similar study by Khajuria reported 68% of isolates from male patients [15]. Other studies by Nimbalkar (59.4%) and Pandya and Yagnesh (74%) also found a higher proportion of male isolates [16-17]. Andhale reported an even higher percentage (76%) of male isolates, exceeding the findings of this study [14].

In the current study, the inpatient department accounted for 87% of the isolates, and the outpatient department accounted for 13%. Similarly, Sreeshma [9] reported 78% and Jayanthi reported 70.94% from the inpatient department, both lower than the present findings [11].

In this study, most isolates (28.86%) were acquired from the surgery ward, followed by ICUs (27.96%). This aligns with the studies of Jayanthi [11] and Khajuria, which reported 32% of isolates from the surgery ward [15]. However, Sreeshma found a higher rate of 44% from the surgery ward [9].

Fifty percent of the isolates were from the urine samples, consistent with the findings of Mohan (35%) [18]. On the other hand, Amsaveni reported 32% of isolates from urine samples [19], while Goyal observed 22% [20].

Antimicrobial drugs were tested against the isolated bacterial strains, and higher resistance to imipenem (100%), followed by piperacillin (97.5%), levofloxacin (94.9%), meropenem (94.1%), ciprofloxacin (93.2%), piperacillin-tazobactam (91.5%), ceftazidime (83.9%), gentamicin (80.50%), amikacin (73.7%), and cefepime (68.7%) was observed. Mohite reported maximum resistance to imipenem (100%), followed by ceftazidime (100%), ciprofloxacin (92.30%), gentamicin (87.91%), piperacillin-tazobactam (85.71%), piperacillin (76.82%), and amikacin (75.82%) [21]. These findings are relatable to the reported present study. In contrast, the findings of Chourasia showed maximum resistance to gentamicin (61.53%), followed by ciprofloxacin (44.61%), imipenem (44.61%), and meropenem (44.61%) [13]. According to Chauhan, 81% of the isolates showed resistance to gentamicin, 67.5% to cefepime, 66% to amikacin, 53% to ceftazidime, and only 36.5% to imipenem [12]. As per ElMasry et al. and Bharti, the isolates observed to be resistant to imipenem were 71% and 34.41%, respectively, which were lower in comparison to the present study [22-23].

A significant limitation of the present study was the lack of investigation into the minimum inhibitory concentration (MIC) breakpoints of the isolates, as well as a detailed molecular analysis. Investigating MIC breakpoints is crucial for understanding the effectiveness of antibiotics against the isolates, while molecular analysis could have provided insights into the genetic characteristics and resistance mechanisms of the pathogens. It is recommended that future research initiatives incorporate quantitative polymerase chain reaction assays to accurately assess and confirm variations in the expression of different resistance genes in MDR *Pseudomonas aeruginosa*.

## Conclusions

The findings of our study revealed a rising demand for consistent monitoring of antibiotic resistance. This is crucial to detect and track the development of resistance patterns in bacteria, especially in hospital environments where the spread of MDR strains poses a significant threat. To combat this, it is essential to not only regularly surveil resistance but also enhance and reinforce infection control measures. This involves rigorous hygiene practices, isolation protocols, and other preventive strategies to stop the transmission of these dangerous bacteria.

Moreover, an effective antibiotic stewardship program is critical. Such a program promotes the responsible use of antibiotics by ensuring they are prescribed only when necessary and in the correct doses. Along with this, a strict antibiotic policy must be implemented, enforcing guidelines on the use of antibiotics to prevent overuse and misuse. These actions are critical for both controlling the current infections and preventing the evolution of further resistance, making them an urgent necessity in hospital settings to protect patient’s health and public safety.
